# Multiplex Gene Expression Profiling of 16 Target Genes in Neoplastic and Non-Neoplastic Canine Mammary Tissues Using Branched-DNA Assay

**DOI:** 10.3390/ijms17091589

**Published:** 2016-09-21

**Authors:** Florenza Lüder Ripoli, Susanne Conradine Hammer, Annika Mohr, Saskia Willenbrock, Marion Hewicker-Trautwein, Bertram Brenig, Hugo Murua Escobar, Ingo Nolte

**Affiliations:** 1 Small Animal Clinic, University of Veterinary Medicine Hannover, Hannover D-30559, Germany; florenza@ripoli.com.br (F.L.R.); Susanne.Conradine.Hammer@tiho-hannover.de (S.C.H.); annika.mohr@tiho-hannover.de (A.M.); willenbrock@preclinics.com (S.W.); 2 Hematology Oncology and Palliative Medicine, Clinic III, University of Rostock, Rostock D-18057, Germany; hugo.murua.escobar@med.uni-rostock.de; 3 Department of Pathology, University of Veterinary Medicine Hannover, Hannover D-30559, Germany; marion.hewicker-trautwein@tiho-hannover.de; 4 Institute of Veterinary Medicine, Georg-August-University Göttingen, Göttingen D-37077, Germany; bbrenig@gwdg.de

**Keywords:** canine mammary tumor, gene expression, RNA, multiplexed branched-DNA (b-DNA) assay, formalin-fixed, paraffin-embedded samples, fresh frozen tissue

## Abstract

Mammary gland tumors are one of the most common neoplasms in female dogs, and certain breeds are prone to develop the disease. The use of biomarkers in canines is still restricted to research purposes. Therefore, the necessity to analyze gene profiles in different mammary entities in large sample sets is evident in order to evaluate the strength of potential markers serving as future prognostic factors. The aim of the present study was to analyze the gene expression of 16 target genes (*BRCA1*, *BRCA2*, *FOXO3*, *GATA4*, *HER2*, *HMGA1*, *HMGA2*, *HMGB1*, *MAPK1*, *MAPK3*, *MCL1*, *MYC*, *PFDN5*, *PIK3CA*, *PTEN*, and *TP53*) known to be involved in human and canine mammary neoplasm development. Expression was analyzed in 111 fresh frozen (FF) and in 170 formalin-fixed, paraffin-embedded (FFPE) specimens of neoplastic and non-neoplastic canine mammary tissues using a multiplexed branched-DNA (b-DNA) assay. *TP53*, *FOXO3*, *PTEN*, and *PFDN5* expression revealed consistent results with significant low expression in malignant tumors. The possibility of utilizing them as predictive factors as well as for assisting in the choice of an adequate gene therapy may help in the development of new and improved approaches in canine mammary tumors.

## 1. Introduction

Approximately 50% of canine mammary tumors appear to be malignant [1,2]. Certain breeds are reported to be predisposed with influence of the geographic location [3]. The highest relative risk ratio of developing benign mammary tumors was found in Yorkshire terriers followed by poodles, dachshunds, and cocker spaniels in Germany. In malignant mammary tumors, the prevalence was higher in poodles followed by dachshunds, cocker spaniels, and Yorkshire terriers [4].

The postoperative median survival time can be shorter than two years depending on the clinical stage and histologic grade of the tumor [5]. Therefore, the clinical stage at mammary tumor diagnosis is one of the most important prognostic factors in dogs [6]. However, it is only possible to be assessed in symptomatic patients and is thus not suitable as a mammary tumor predictive parameter.

Immunohistochemistry (IHC), alongside conventional histopathology, plays an important role as a diagnostic tool in classifying tumors in humans and dogs [7,8]. Most studies on mammary tumors in dogs are made through IHC and determine biomarkers on the protein level [9]. This technique has proven to be suitable when performing large multicenter studies. However, the limitations rely on the semiquantitative and subjective interpretation of its results [10]. Therefore, the present study utilized branched DNA (b-DNA) assay which, in combination with xMAP^®^ magnetic beads technology, permits the concomitant expression analysis of several target genes within a single sample [11]. It also presents a simpler and more rapid workflow, providing highly sensitive quantitative results when compared to other gene expression techniques such as real-time polymerase chain reaction (qPCR) [12], which is commonly the method of choice when analyzing gene expression [13]. The possibility of analyzing a vast number of samples at the same time is also an important advantage of the proposed technique.

The need to comprehensively analyze gene profiles in different canine mammary tumor (CMT) entities to find potential candidate genes which could serve as predictive factors is evident, considering little is known in this regard to date [14]. Thus, the detection of suitable biomarkers could assist in the neoplasms′ early detection and support in providing a therapeutic approach.

In contrast to the human counterpart, the use of mammary tumor biomarkers in canines is still restricted to research purposes [14,15]. Expression of *BRCA1* and *BRCA2* in CMT was analyzed and it was found to be associated with an increased mammary tumor risk in English Springer Spaniels (ESS) [16]. A possible influence of those genes in tumor development is also suggested in other studies [17,18,19,20]. Further targets of those analyzed herein have also been investigated in CMT such as *TP53* [21,22], *PTEN* [23,24,25,26,27], *PFDN5* [15,26], *MYC* [27], *HER2* [28], and *MCL1* [29] associating their expression patterns with the development of CMT.

The present study proposes the concomitant analysis of 16 onco- and suppressor-genes (*BRCA1*, *BRCA2*, *FOXO3*, *GATA4*, *HER2*, *HMGA1*, *HMGA2*, *HMGB1*, *MAPK1*, *MAPK3*, *MCL1*, *MYC*, *PFDN5*, *PIK3CA*, *PTEN*, and *TP53*) regarded as being involved in neoplasm development using multiplex branched-DNA technology in fresh frozen (FF) and formalin-fixed, paraffin-embedded (FFPE) tissues. A previous study demonstrated that the analysis of canine mammary FF and FFPE samples via b-DNA assay is feasible [30]. Therefore, the expression analysis in 111 FF and 170 FFPE specimens of canine mammary tissues aims to compare the expression patterns of the candidate genes in neoplastic (benign and malignant) and non-neoplastic tissues with the intention of finding new potential tumor markers. This characterization might serve to gain a better understanding of the tumor pathogenesis of different canine mammary tumor types in further studies. Moreover, potential target genes might contribute to determining whether certain breeds are at risk of developing mammary tumors and are able to contribute to the therapeutic approach.

## 2. Results

Of the 180 FFPE specimens, 15 of them (7 benign tumors and 6 malignant tumors) were entirely excluded due to values below the LOD (limit of detection). Ninety-eight of FFPE samples of *GATA4* were excluded because of the LOD, whereas 84 FFPE samples of *HMGA2* were excluded. *BRCA1* also showed a high number of exclusions with 63 FFPE samples. The remaining samples of *GATA4*, *HMGA2*, and *BRCA1* as well as all specimens of *MYC*, *BRCA2*, and *MAPK1* in both FF and FFPE samples revealed low expression (below 0.1) and no significant differences were found between the histologic groups.

**Figure 1:**
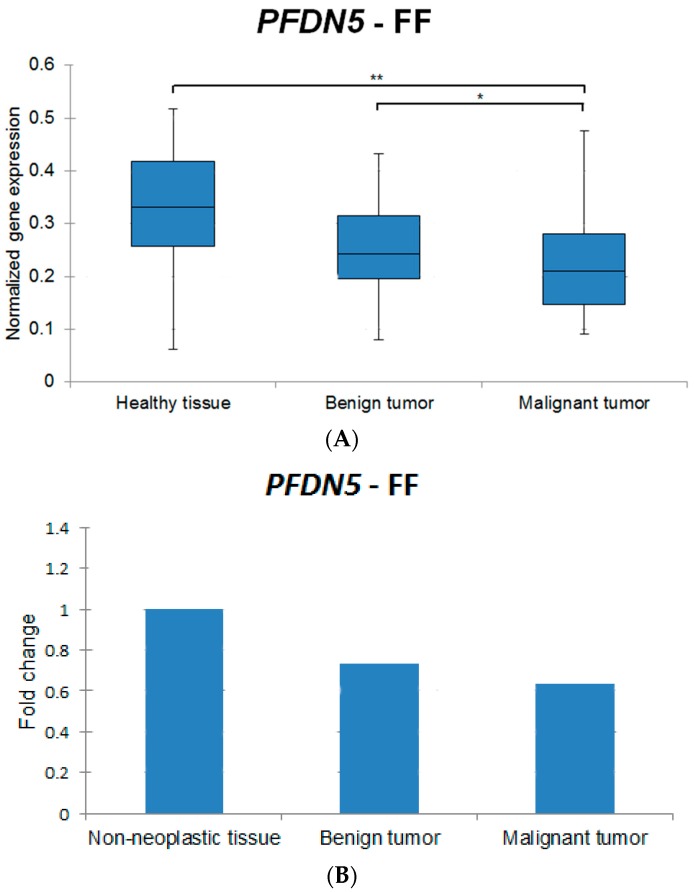
(**A**) Exemplary box plot indicating the normalized gene expression of *PFDN5* in fresh frozen samples in the different histologic groups. Statistically significant differences are indicated by an asterisk (*). Results are classified as significant (* *p* < 0.05) and very significant (** *p* < 0.01). The box includes cases from the 25th to the 75th percentile. The horizontal line within the box represents the median and the upper and lower bars are the largest and lowest observed values. Significant differences are revealed between the groups healthy tissue vs. malignant tumors and benign tumors vs. malignant tumors; (**B**) Normalized expression fold changes (mean) indicating the percentage change of *PFDN5* in fresh frozen samples in the different histologic groups.

**Figure 2:**
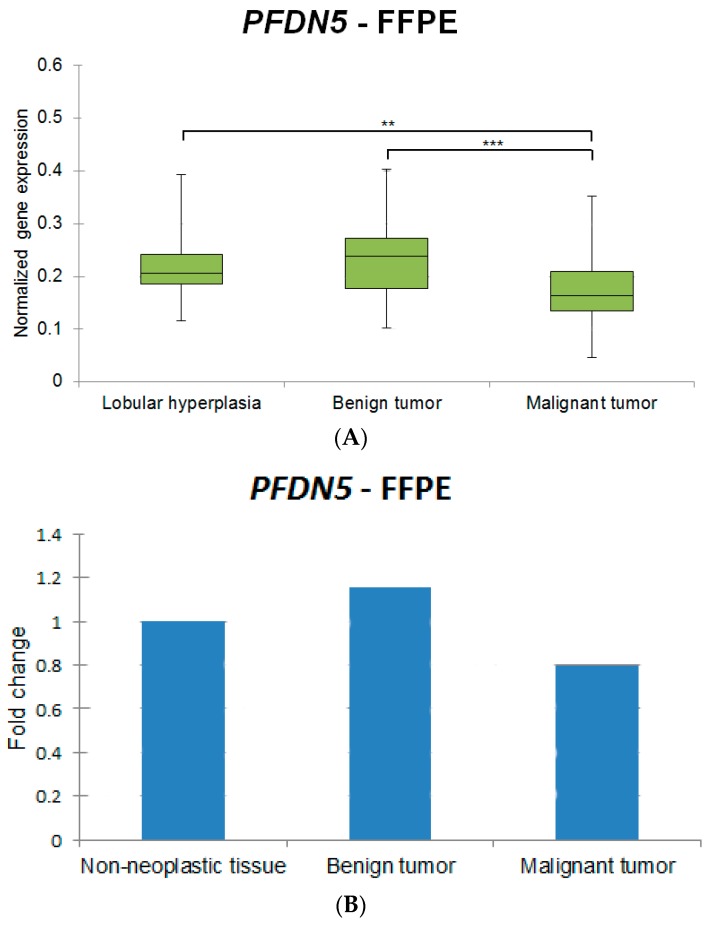
(**A**) Exemplary box plot indicating the normalized gene expression of *PFDN5* in formalin-fixed, paraffin-embedded samples in the different histologic groups. Statistically significant differences are indicated by an asterisk (*). Results are classified as very significant (** *p* < 0.01) and extremely significant (*** *p* < 0.001). The box includes cases from the 25th to the 75th percentile. The horizontal line within the box represents the median and the upper and lower bars are the largest and lowest observed values. Significant differences are revealed between the groups lobular hyperplasias vs. malignant tumors and benign tumors vs. malignant tumors. A lower level of expression can be clearly identified when comparing the expression of *PFDN5* in fresh frozen samples (Figure 1); (**B**). Normalized expression fold changes (mean) indicating the percentage change of *PFDN5* in formalin-fixed, paraffin-embedded samples in the different histologic groups.

All genes from FFPE samples except for *PTEN* showed lower expression levels when compared to FF specimens (exemplary box plots for the gene *PFDN5* are shown in Figure 1A and Figure 2A, where different expression levels between FF and FFPE specimens are noted). The samples of FF and FFPE origin were analyzed separately.

Exemplary graphs representing the percentage change of *PFDN5* between the histologic groups in FF and FFPE are represented in Figure 1B and Figure 2B, respectively.

The expression of the housekeeping genes (HKG) varied, and the mean expression of the three housekeeping genes was calculated to achieve a more accurate normalization.

The number of samples in each group are listed in Table 1.

### 2.1. Gene Expression

#### 2.1.1. Fresh Frozen Tissues

Table 2 shows the results of the target gene expression in FF samples when comparing the different histologic groups. The group of lobular hyperplasias was excluded due to the small number of samples (*n* = 3). The non-neoplastic tissue for FF specimens was considered to be healthy mammary tissue.

In the groups healthy tissue vs. benign tumors and healthy tissue vs. malignant tumors, all target genes with statistically significant differences showed higher expression in the healthy mammary tissue. In benign tumors vs. malignant tumors, all genes were more highly expressed in benign tumors (Table 2).

#### 2.1.2. Formalin-Fixed, Paraffin-Embedded Tissues

Table 3 summarizes the results of the target gene expression in FFPE samples when comparing the different histologic groups. The group of healthy mammary tissue was excluded due to the insufficient number of samples (*n* = 4). The group lobular hyperplasia was considered as the non-neoplastic tissue for FFPE specimens. Results of lobular hyperplasias vs. benign tumors are not displayed in the table due to the lack of statistically significant differences.

All genes showing statistically significant differences in lobular hyperplasias vs. malignant tumors were more highly expressed in lobular hyperplasias (non-neoplastic tissue). Between the groups benign tumors vs. malignant tumors, all genes revealing statistically significant differences were more highly expressed in the benign tumors.

## 3. Discussion

The gene expression profile of the mammary tumor in dogs is, to date, not well characterized and the use of tumor markers is still for research purposes only [15]. The analysis and differentiation of the mammary tumors is the basis for pathogenesis, diagnosis, prognosis, new therapy options, and good breed hygiene practice. Until now, most mammary studies in dogs have utilized IHC and analyzed proteins [9]. IHC has proved to be an excellent technique when performing large multicenter studies. Nonetheless, it is laborious and time-consuming when analyzing several target genes [31]. The present study proposes the analysis of 16 target genes using branched-DNA assay which, when allied with xMAP^®^ magnetic beads, enables the analysis of several genes within a single sample (multiplexing).

The main aim of this study was to compare the expression of target genes known to be involved in tumor growth in dogs and humans [32,33,34] in different FF and FFPE canine mammary samples, as well as to find new potential tumor markers.

The significant reduction in the expression of the tumor suppressors *TP53*, *FOXO3*, *PTEN*, and *PFDN5* in malignant tumors found in the present study confirms previous reports [35,36,37,38] and underlines their role in cellular growth. Hence, these genes could represent potential markers to predict CMT.

To the best of our knowledge, no specific reports on the expression of *FOXO3* in canine mammary tissues have been elucidated so far. The results of the present study corroborate what has already been reported in humans, demonstrating its higher expression in non-neoplastic tissues. The inactivation of FOXO subfamily controls several functions, including cell differentiation, proliferation, cell death, metabolism, and longevity [35]. A study from 2013 revealed that a high expression level of *FOXO3* was significantly correlated with long-term survival, indicating that *FOXO3* expression is a favorable prognostic marker in breast cancer [39] and this may also be applicable for dogs.

In the present study, *TP53* was revealed to be more highly expressed in non-neoplastic tissue. However, most of the existing studies on humans and canines analyzed point mutations instead of gene expression levels [21,22,36,40]. Muto and colleagues revealed that mutations were detected not only in mammary carcinomas but also in benign tumors [21]. To the best of our knowledge, no gene expression analysis has been performed with *TP53* in dogs to date. Thus, it is suggested that the results herein might correlate with existing reports [36,41] where the described presence of mutations could correspond to the lower levels of *TP53* in neoplastic tissues found in our study. Moreover, *TP53* is known to be a tumor suppressor gene [42] and, therefore, its lower expression in neoplastic tissues might lead to uncontrolled cellular growth, a fact which corroborates the findings herein. Further studies at mRNA level are worth carrying out to deepen existing knowledge concerning the role of this gene in CMT.

The present study revealed higher expression of *PTEN* in non-neoplastic tissues and benign tumors in FFPE samples, thus supporting existing studies. *PTEN* is known as a tumor suppressor gene [37] and the role of its loss has been largely investigated in human breast carcinomas [43,44,45]. Known for its importance in humans, the role of *PTEN* has also been investigated in CMT and its behavior in dogs resembles what has been seen in humans, associating low expression of *PTEN* with malignancy [23,24,25,26]. A recent study revealed frequent loss of the region harboring *PTEN* on the canine chromosome 26, indicating an important event in CMT development [27].

Rare reports mention the role of *PFDN5* in breast cancer. However, it has been shown to be a tumor suppressor candidate in leukemia and tongue cancer [38]. The outcomes herein revealed higher expression in non-neoplastic tissue, suggesting it as a potential tumor marker just as demonstrated by Hennecke and colleagues [15]. Moreover, a previous study demonstrated that *PFDN5* was recurrently deleted in CMT [26].

The genes *MAPK3*, *HMGB1*, *HMGA1*, *PIK3CA*, and *MCL1* are genes known to stimulate cellular growth; however, contrary to what is normally reported in humans [46,47,48,49,50], in the present study these genes were shown to be consistently expressed at lower levels in malignant tumors. Therefore, it is hypothesized that those genes play a different role in canine mammary tumors.

The role of *HER2* is well characterized in humans and it is widely used as a prognostic factor [51,52]. Interestingly, in contrast to a previous study on CMT which revealed different percentages of *HER2* being overexpressed in carcinomas [28], the present study demonstrated lower expression of the referred gene in malignant tumors. The relevance of its overexpression as a prognostic factor has, however, still not been clearly determined [28]. Therefore, further analyses are necessary for a better understanding of the interaction of *HER2* in CMT.

Intriguingly, the present study did not show conclusive results for *BRCA1* and *BRCA2* despite their well-characterized role and importance in human [53] and canine [16] mammary tumors. The reason for this could be the extremely low expression of both genes in the FF and FFPE samples investigated here.

## 4. Materials and Methods

### 4.1. Tissue Samples

#### 4.1.1. Formalin-Fixed, Paraffin-Embedded Blocks

The samples utilized in this work were retrospectively retrieved from the Department of Pathology, University of Veterinary Medicine Hannover, Hannover, Germany, between the years 1993 and 2000. The mammary specimens were fixed in 10% neutral buffered formalin as routinely performed and embedded in paraffin wax. The blocks were stored in the archive at room temperature. A total number of 170 samples were selected for the analysis.

#### 4.1.2. Fresh Frozen Tumor Samples

The samples were obtained from patients which had undergone surgery in the Small Animal Clinic, University of Veterinary Medicine Hannover, Hannover, Germany, between 2003 and 2011 (identification number: 96A697; 19 January 2005), as well as from patients from the Clinic of Small Animals, Institute of Veterinary Medicine, Georg-August-University Göttingen, Göttingen, Germany, from 2013 and 2014. All utilized tissue samples were removed with consent of the owner. The specimens were frozen in liquid nitrogen directly after surgical removal and maintained at −80 °C until RNA isolation. A total number of 111 samples were selected for the analysis.

### 4.2. Nucleic Acid Isolation and Quantification

#### 4.2.1. Formalin-Fixed, Paraffin-Embedded Samples

Prior to RNA isolation, two 20 µm thick paraffin sections of the FFPE blocks were prepared using a microtome (pfm Slide 2003, pfm medical AG, Cologne, Germany). Samples were deparaffinized using the Deparaffinization Solution (Qiagen GmbH, Hilden, Germany) and the RNA was isolated as previously described [30]. Samples were stored at −80 °C until the experiment required further usage.

#### 4.2.2. Fresh Frozen Samples

Prior to the nucleic acid isolation, frozen tissue was homogenized using a Tissue Lyser II and 5 mm stainless steel beads (Qiagen GmbH). Nucleic acid isolation was performed with the RNeasy Mini kit (Qiagen GmbH) following the manufacturer’s instructions, with an additional step of digestion using the RNase-free DNase Set (Qiagen GmbH) according to the manufacturer’s protocol. Additionally, genomic DNA was digested using RQ1 RNase-free DNase (Promega GmbH, Mannheim, Germany). RNA yield was determined with the Take3 Micro-Volume Plate (BioTek, Bad Friedrichshall, Heilbronn, Germany). Samples were stored at −80 °C until use.

#### 4.2.3. Target Genes

The following 16 canine genes were analyzed: *BRCA1*, *BRCA2*, *FOXO3*, *GATA4*, *HER2*, *HMGA1*, *HMGA2*, *HMGB1*, *MAPK1*, *MAPK3*, *MCL1*, *MYC*, *PFDN5*, *PIK3CA*, *PTEN*, and *TP53* (Table 4).

#### 4.2.4. Housekeeping Genes

Three different housekeeping genes (HKG) were used: *ACTB* (β-actin), *GAPDH* (glyceraldehyde 3-phosphate dehydrogenase), and *HPRT1* (hypoxanthine phosphoribosyltransferase 1) (Table 4).

#### 4.2.5. Multiplexed Branched-DNA (b-DNA) Assay

Gene expression was analyzed using the QuantiGene 2.0 Plex Assay (Affymetrix, Santa Clara, CA, USA). Probe sets for the target genes were custom designed based on their accession numbers (Table 4) from Affymetrix.

Briefly, each bead set is coated with a reagent specific to a certain target in order to detect a special analyte in the analyzed material. Multiple readings are made on each bead set resulting in an individual fluorescent signal for each assay. The multiplex branched-DNA assay permits rapid and accurate analysis of up to 100 targets within a single specimen.

In preliminary experiments, an evaluation of the technology on the biological material was performed in order to identify the ideal sample input for the specimens. These analyses revealed that 250 and 200 ng of RNA were sufficient amounts to perform the assay for FF and FFPE specimens, respectively. Moreover, considering that the amount of duplicate measurements of certain samples intra-assay were consistent (represented by a low coefficient of variation), a single measurement was considered sufficient. The assay was carried out according to the manufacturer’s protocol.

### 4.3. Histologic Classification

All samples were evaluated and diagnosed using hematoxylin and eosin staining. Samples were separated into the following four groups: (1) healthy mammary tissue (non-neoplastic tissue); (2) lobular hyperplasia (non-neoplastic tissue); (3) benign tumor; and (4) malignant tumor.

### 4.4. Data and Statistical Analysis

Specimens with expression values below the limit of detection (LOD—background plus 3 times standard deviation of the background) were excluded (according to recommendations by the assay provider). Samples were normalized against the average of the three HKG. Data were not normally distributed and therefore the Mann-Whitney *U* test was performed using the software SAS Enterprise Guide 7.1 (SAS Institute Inc., Cary, NC, USA). Results were classified as significant (* *p* < 0.05), very significant (** *p* < 0.01), and extremely significant (*** *p* < 0.001).

## 5. Conclusions

In conclusion, based on the outcomes of the present study, the suppressor genes *TP53*, *FOXO3*, *PTEN* and *PFDN5* are considered as potential markers for predicting canine mammary tumors. The results herein are in line with the literature, revealing low expression of these genes in malignant tumors, suggesting they play a role in cellular growth. Further investigations are needed to prove if these genes alone play a role in general tumor progression or if they, in combination with other genes, confer an important predisposition to the disease with further development of mammary tumors. Moreover, the clinical follow-up data of the patients would assist in the interpretation of the gene expression results herein described. A comparison of the expression between already existing methods for gene expression measurements and multiplex branched-DNA assay would enable the establishment of a cutoff for each target gene in the different histologic groups.

The multiplex technology is certainly a method which enables the analysis of several samples which present small amounts of starting material of FF and FFPE tissue. Moreover, several target genes are concomitantly analyzed, thereby reducing hands-on time and costs.

## Figures and Tables

**Table 1:** Number of specimens of fresh frozen (FF) and formalin-fixed, paraffin-embedded (FFPE) per group.

Group	FF (*n*)	FFPE (*n*)
1. Healthy mammary tissue (non-neoplastic tissue)	15	4
2. Lobular hyperplasias (non-neoplastic tissue)	3	20
3. Benign tumors	33	47
4. Malignant tumors	60	84
Total	111	155

**Table 2:** Gene expression of the target genes when comparing the different canine mammary tissues from FF samples. Arrows show a higher or lower expression of the first group in relation to the second one (example: first group vs. second group).

Target Gene	Healthy Tissue vs. Benign Tumors	Healthy Tissue vs. Malignant Tumors	Benign Tumors vs. Malignant Tumors
*BRCA1*	-	-	-
*BRCA2*	-	-	-
*FOXO3*	-	↑ *	↑ *
*GATA4*	-	-	-
*HER2*	-	↑ *	↑ *
*HMGA1*	↑ **	↑ *	-
*HMGA2*		-	-
*HMGB1*	↑ *	-	-
*MAPK1*	-	-	-
*MAPK3*	-	↑ *	-
*MCL1*	↑ *	↑ **	↑ *
*MYC*	-	-	-
*PFDN5*	-	↑ **	↑ *
*PIK3CA*	-	-	-
*PTEN*	-	-	-
*TP53*	-	↑ *	-

Results are classified as significant (* *p* < 0.05) and very significant (** *p* < 0.01). Cells with hyphen (-) revealed no significant differences.

**Table 3:** Gene expression of the target genes when comparing the different canine mammary tissues from FFPE samples. Arrows show a higher or lower expression of the first group in relation to the second one (example: first group vs. second group).

Target Gene	Lobular Hyperplasias vs. Malignant Tumors	Benign Tumors vs. Malignant Tumors
*BRCA1*	-	-
*BRCA2*	-	-
*FOXO3*	↑ **	↑ ***
*GATA4*	-	-
*HER2*	↑ ***	↑ ***
*HMGA1*	↑ *	-
*HMGA2*	-	-
*HMGB1*	↑ ***	↑ *
*MAPK1*	-	-
*MAPK3*	↑ **	↑ ***
*MCL1*	↑ ***	↑ ***
*MYC*	-	-
*PFDN5*	↑ **	↑ ***
*PIK3CA*	↑ *	↑ **
*PTEN*	-	↑ **
*TP53*	↑ *	↑ *

Results are classified as significant (* *p* < 0.05), very significant (** *p* < 0.01), and extremely significant (*** *p* < 0.001). Cells with hyphen (-) revealed no significant differences.

**Table 4:** Target and housekeeping genes with their approved name and respective accession numbers.

Target Gene	Approved Name	Accession Number
*BRCA1*	BRCA1, DNA repair associated	NM_001013416
*BRCA2*	BRCA2, DNA repair associated	NM_001006653
*FOXO3*	Forkhead box O3	NM_003639400
*GATA4*	GATA binding protein 4	NM_001048112
*HER2*	Erb-b2 receptor tyrosine kinase 2	NM_001003217
*HMGA1*	High mobility group AT-hook 1	NM_001003387
*HMGA2*	High mobility group AT-hook 2	XM_005625590
*HMGB1*	High mobility group box 1	NM_001002937
*MAPK1*	Mitogen-activated protein kinase 1	NM_001110800
*MAPK3*	Mitogen-activated protein kinase 3	NM_001252035
*MCL1*	Myeloid cell leukemia 1	NM_001003016
*MYC*	V-myc avian myelocytomatosis viral oncogene homolog	NM_001003246
*PFDN5*	Prefoldin subunit 5	NM_001251949
*PIK3CA*	Phosphatidylinositol-4,5-bisphosphate 3-kinase catalytic subunit α	XM_545208.4
*PTEN*	Phosphatase and tensin homolog	NM_001003192
*TP53*	Tumor protein p53	NM_001003210
*ACTB*	β-actin	XM_536888
*GAPDH*	Glyceraldehyde 3-phosphate dehydrogenase	NM_001003142
*HPRT1*	Hypoxanthine phosphoribosyltransferase 1	NM_001003357
